# Dr. Joshua Tucker

**Published:** 1881-12

**Authors:** 


Dr. Joshua Tucker, well known to the citizens of Boston for the last fifty years, died at Winchendom, his native town, on the 7th of November, 1881. Dr. Tucker was born August 7th, 1800. At the age of eighteen he left his native place, and after the pursuit of business for several years, began the study of dentistry with Dr. D. C. Ambler, of Columbia, South Carolina, and continued his studies with Dr. C. Starr Brewster, of Charleston, meanwhile attending lectures at the South Carolina

Medical College. After the completion of his studies he went to Havana, Cuba, where he continued in the practice of his profession for several years, until driven away by the yellow fever, of which he himself was a sufferer. Coming to Boston in 1833, he became associated with Dr. Daniel Harwood, and the names of Harwood and Tucker became well known not only in New England but throughout this country and Europe as well. For many years Dr. Tucker was a great sufferer from facial neuralgia, and in 1853, being much impaired in health, he visited Europe, where he remained two years, employing the best skill that the great capitals afforded, but without permanent relief. On returning home he resumed active practice, which he only relinquished some five or six years since. Coming of a long lived and hardy race, simple and pure in his habits, he fought bavely for renewed health, and for some years past has been comparatively free from suffering, passing peacefully away after a short illness, at the ripe age of 81 years and three months. He was peculiarly genial in temperament, and through all his sufferings retained unbounded hope and faith, and looked upon the cheerful side of the vicissitudes of life. He was in all senses a loveable man, and was deeply loved and highly respected by all who knew him. He was a member of the Massachusetts Medical Society since 1838, and was president and honorary member of numerous professional societies, including the Odontological Society of Great Britain. Dr. Tucker was a member of St. John's Lodge, F. and A. M., and also a charter member of the DeMolay encampment of Knights Templar.

